# Draft Genome Sequence of *Magnetospirillum* sp. Strain SO-1, a Freshwater Magnetotactic Bacterium Isolated from the Ol’khovka River, Russia

**DOI:** 10.1128/genomeA.00235-14

**Published:** 2014-04-10

**Authors:** Denis S. Grouzdev, Marina V. Dziuba, Marina S. Sukhacheva, Andrey V. Mardanov, Aleksey V. Beletskiy, Boris B. Kuznetsov, Konstantin G. Skryabin

**Affiliations:** a Centre Bioengineering RAS, Moscow, Russia; b Faculty of Biology, Lomonosov Moscow State University, Moscow, Russia

## Abstract

Here, we present the draft genome sequence of *Magnetospirillum* sp. strain SO-1, a freshwater magnetotactic spirillum isolated from the sediments of the Ol’khovka River, Russia.

## GENOME ANNOUNCEMENT

Magnetotactic bacteria are phylogenetically diverse microorganisms capable of synthesizing well-organized nanosized magnetic crystals enveloped by membrane, referred to as magnetosomes. The magnetosome biomineralization is under strict genetic control, and the genes responsible for a magnetic phenotype are combined in the magnetosome genomic island (MAI) (1, 2). Here, we present the draft genome sequence of *Magnetospirillum* sp. strain SO-1, a freshwater magnetotactic spirillum isolated from the sediments of the Ol’khovka River (43°56′07″N, 42°41′25″E; Caucasus region, Russia) (3).

The genome sequencing of *Magnetospirillum* sp. SO-1 was carried out using 454 GS FLX technology. Sequencing resulted in 284,391 reads, with an average read length of 356 bp and approximately 17× coverage. The reads were assembled with GS *De Novo* Assembler 2.6 and generated 261 contigs, with an *N*_50_ of 55,386 bp. The draft genome of *Magnetospirillum* sp. SO-1 consists of 261 contigs of 4,874,064 bp, with an average G+C content of 65.98 mol%. According to the primary annotation performed through the NCBI Prokaryotic Genomes Automatic Annotation Pipeline (PGAAP) (4), the genome contains 3 rRNA genes (5S-23S-16S) and 52 aminoacyl-tRNA synthetase genes. The genome coding density is 89.98%, with an average gene length of 941 bp. A total of 4,717 coding regions were found in the genome, of which 3,573 (75.75%) were functionally annotated using Rapid Annotations Subsystems Technology (RAST) online service (5). The genes essential for magnetosome formation in *Magnetospirillum* sp. SO-1 were organized in four operons within the MAI (*mamGFDC*, *mms6*, *mamAB*, and *mamXY*), as was shown previously for other *Magnetospirillum* spp. (2). The estimated size of the MAI is approximately 100 kb.

A functional comparison of the genome sequences available on the RAST server revealed the closest neighbors of *Magnetospirillum* sp. SO-1 to be *Magnetospirillum magneticum* AMB-1 (score, 548), *Magnetospirillum gryphiswaldense* MSR-1 (score, 527), *Rhodospirillum rubrum* ATCC 11170 (score, 408), and *Azospirillum* sp. strain B510 (score, 259).

### Nucleotide sequence accession numbers.

This whole-genome shotgun project has been deposited at DDBJ/EMBL/GenBank under the accession no. AONQ00000000. The version described in this paper is the first version, AONQ01000000.
